# Intention to disclose maternal HIV status to children among Chinese mothers: association with outcome expectations and moderation by perceived health

**DOI:** 10.1186/s12889-025-23042-2

**Published:** 2025-05-23

**Authors:** Qian Wang, Yinghui Sun, Ho Hin Lee, Kam Hei Hui, Ailing Wang, Xiaoyan Wang, Xinwei Li, Phoenix Kit-Han Mo

**Affiliations:** 1 National Center for Women and Children’s Health, NHC, PRC (NCWCH), Beijing, China; 2 Centre for Health Behaviours Research, JC School of Public Health and Primary Care, The Chinese University of Hong Kong, Hong Kong, China; 3 The Chinese University of Hong Kong Shenzhen Research Institute, Shenzhen, Guangdong China

**Keywords:** HIV status, Disclosure intention, Women, Outcome expectations, Perceived health

## Abstract

**Background:**

Disclosure of maternal HIV status to children can be beneficial for both mother and children. Few studies have examined factors and moderators of HIV disclosure among mothers living with HIV.

**Objective:**

The present study examined the association of outcome expectations and perceived health on intention to disclose maternal HIV status to children, and the moderating role of perceived health among women living with HIV in China.

**Method:**

A cross-sectional survey was conducted among 269 women with HIV who had at least one living child aged > 5 years and had not yet disclosed their HIV status to their oldest child in China.

**Results:**

Results from hierarchical regression revealed that after adjusting for significant socio-demographic and medical variables, positive outcome expectations had a positive association and negative outcome expectations had a negative association with intention to disclose HIV. Perceived health had no significant association with intention to disclose HIV. Furthermore, a significant moderation effect of perceived health on positive outcome expectations was found. Further analysis showed that the association of positive outcome expectations and intention to disclose HIV was stronger among women with better perceived health.

**Conclusion:**

Findings support the importance of outcome expectations, and the need to consider the moderating role of perceived health on HIV disclosure among women living with HIV in China.

## Background

According to the UNAIDS, China had an estimated 1.3 million people living with HIV who know their status by the end of 2023 [1]. Despite an increase in HIV infections and AIDS-related deaths, the mortality rate has significantly decreased from 10.9% in 2007 to 4.3% in 2019, with an average time from diagnosis to death lengthening from 1.4 to 4.0 years due to improved antiviral treatment [2]. Meanwhile, the HIV transmission pattern has shifted, with heterosexual and homosexual transmission becoming more prominent. A refinement of key populations becomes indispensable for the evaluation of further intervention strategies.

### Maternal self-disclosure of HIV status

Women represent an important vulnerable group for HIV in China. With the advances in HIV prevention and treatment, most children born to women with HIV are HIV-uninfected. As more women with HIV live healthy and continue their roles as mothers and caregivers, supporting them in disclosing their HIV status to their HIV-uninfected children becomes a paramount public health issue. Self-disclosure involves the deliberate sharing of previously concealed, potentially stigmatizing information, aiming to strengthen relationships and enhance psychological well-being for both discloser and recipients [3, 4] With reference to WHO recommendations, parents or caregivers should disclose their HIV status to school-age children, using age-appropriate communication tailored to the child’s cognitive and emotional development [5].

Research has summarized numerous benefits of maternal HIV disclosure, particularly in strengthened parent-child relationships and reduced psychological burden [6, 7]. Disclosure alleviates the stress of maintaining HIV status secrecy and the fear of unintentional disclosure [8], fostering improved family bonds and honest communication [9–11]. Additionally, maternal HIV disclosure has been shown to positively impact children’s mental and social well-being [7, 12, 13]. As HIV progression heightens the need for disclosure, these benefits underscore its importance in shaping HIV care strategies and advancing public health goals.

Nevertheless, the self-disclosure level among women living with HIV remains low. Systematic reviews from Qiao et al. [8] found disclosure rates ranging from 20 to 67% in the U.S. and 11–50% internationally. In China, only 16.8% of women living with HIV intended to disclose their status to the eldest child [14–16], and a study from children’s perspective revealed that only 39% of informed children acknowledged maternal HIV status from their mothers [17]. Moreover, many HIV disclosures were unintentional, occurring when children overheard conversations, or learned indirectly from non-family members [17].

### Perceived health in HIV disclosure

Amongst theoretical frameworks for HIV disclosure, Babcock’s Disease Progression Theory identifies advanced disease progress as a key driver of disclosure intention [18]. As HIV progressed, symptoms of health deterioration, hospitalization, ART uptake and involvement in HIV support services necessitate explanations to family members, making concealment difficult [8, 16]. Studies consistently revealed that parental HIV disclosure significantly increased as health deteriorated or among parents who reported more severe diagnoses [16, 19–21].

### Outcome expectations in HIV disclosure

Outcome expectations refer to individuals’ beliefs about the consequences of engaging in specific behaviors, such as HIV disclosure, and shape their disclosure intentions or decisions. Positive outcome expectations often include improved parent-child relationships and better adjustment and planning for the child. Conversely, negative outcome expectations may involve concerns about strained family relationship and adverse effects on child’s well-being and development [14, 22, 23]. These appraisal processes align with Lazarus and Folkman’s Stress and Coping theory, where outcome expectations reflect cognitive evaluations that influence coping behaviors [24]. A recent study among Chinese women with HIV demonstrated a negative association between negative outcome expectations and maternal HIV disclosure intention [14], highlighting the importance of outcome expectations in shaping disclosure-related decision-making within this theoretical framework.

### The moderating role of perceived health

The Consequence Theory emphasized how anticipated outcomes influence HIV disclosure decisions when perceived benefits outweigh costs, suggesting that disease progression affects disclosure by varying the perceived psychological, social and material outcomes [8]. Therefore, it would be intriguing to explore how perceived health and outcome expectations relate and interact with each other with regards to maternal HIV disclosure. Individuals in better health often approach HIV disclosure more cautiously, whereas those experiencing deteriorating health are more inclined to seek support [8, 16]. It is therefore expected that the effect of outcome expectations would be more relevant among individuals with better health status.

## Methods

### Study design

A cross-sectional survey study was conducted between November 2019 to January 2020. Women living with HIV who had not disclosed their HIV status to their child were targeted. Inclusion criteria were (i) women, (ii) 18 years and above, (iii) Chinese, (iv) diagnosed with HIV, (v) had at least one child over 5 years old, (vi) had not yet disclosed their HIV status to her oldest child. The use of 5 years old as a cut-off has been used in other studies of HIV disclosure [25].

Participants were recruited from 8 Women’s Health Department in Yunnan and Guangxi provinces in mainland China through convenience sampling. Yunnan and Guangxi are located in southwest of China and are geographically adjacent. Both of them have a significant rural population (21.7 million in Guangxi and 22.0 million in Yunnan) [26] and are home to significant ethnic minority (38 in Guangxi and 33% in Yunnan), reflecting comparable cultural and demographic profiles. Eligible participants were identified and invited by the staff and those who expressed initial interest were referred to the research staff. Participants were ensured that they joined the study on a voluntary basis and they could withdraw the study anytime they wished. After obtaining informed consent, a structured interview was conducted by the research staff at the clinic. A standardized protocol was used across all study sites to minimize variability in data collection procedure. A total of 269 participants fulfilled the criteria and were recruited for the study.

### Measures

Socio-demographic information, including age, ethnicity, education level, marital status, monthly family income, number of children, and age and gender of the eldest child were obtained. Disease-related characteristics, such as duration of HIV diagnosis, route of HIV diagnosis, and disease stage were also obtained.

Perceived health was assessed using a single item. Participants were asked to self-rate their own health on a 5-point Likert Scale from 1 = very poor to 5 = very good. Such single item has been commonly used to assess perceived health in many studies [27, 28].

Outcome expectations for HIV disclosure was measured by the Outcome Expectation for Maternal HIV Disclosure Scale previously developed by the team [14]. It measures both positive outcome expectations and negative outcome expectations of HIV disclosure. The positive outcome expectations scale measures 4 dimensions: psychological relief (3 items), improved intimacy (3 items), better planning of the child (3 items), and better adjustment of the child (3 items). The negative outcome expectations scale measures 3 dimensions: negative impact on the parent–child relationship (5 items), negative impact on the child well-being (4 items), and negative impact on the child development (4 items). Participants estimated how likely each outcome would occur if they disclose their HIV status to their eldest child on a 5-point Likert Scale from 1 = very unlikely to 5 = very likely. A higher score reflects higher level of positive and negative outcome expectations, respectively. The Cronbach’s alpha was 0.96 and 0.93 in the present study.

Intention to disclose HIV. Participants were asked to indicate their likelihood to disclose their HIV status to their eldest child in the future on a 5-point Likert Scale from 1 = high unlikely to 5 = highly likely.

### Ethics approval and consent to participate

This study was approved by the Survey and Behavioral Research Ethics Committee of the Chinese University of Hong Kong (SBRE-18-431) and was conducted with the ethical principles outlined in the Declaration of Helsinki. Informed consent was obtained from all participants before the administration of the structured interview.

### Data analysis

Descriptive statistics were reported. Zero-order correlations among the variables were examined to assess the strength and direction of relationships among the variables and to check for potential multicollinearity. A correlation coefficient of 0.7 or higher among two variables would be considered having the problem of potential multicollinearity. Hierarchical regression analyses were conducted to test the association between perceived health, positive outcome expectations, negative outcome expectations, and intention to disclose HIV. Socio-demographic variables and medical variables were entered in Block 1, perceived health was entered in Block 2, and positive outcome expectations and negative outcome expectations were entered in Block 3. To examine the moderating effect of perceived health, interaction terms between positive outcome expectations and perceived health, and between negative outcome expectations and perceived health were created. Multiple regression models that included the main effects of perceived health and positive or negative outcome expectations, and the interaction terms were fitted. These interaction terms were included in hierarchical regression models to test whether the strength or direction of the relationship between outcome expectations and disclosure intention varies depending on participants’ perceived health. A significant interaction would indicate that perceived health moderates the association between outcome expectations on disclosure intention. All analyses were conducted using SPSS Statistics 27.

### Sample size calculation

The sample size was determined using G*Power 3.1 for a multiple linear regression analysis with three predictors (perceived health, positive outcome expectations, and negative outcome expectations). Based on a small-to-medium effect size of *f*^2^ = 0.05, α = 0.05, and power = 0.80, the calculation indicated a required sample size of 222 participants. To accommodate potential incomplete responses or missing data, we targeted a final sample size of at least 250 participants.

## Results

### Background characteristics of the participants

More than two-third of the participants (67.6%) were 40 years old or below. Majority of them were from the Han ethnicity (88.8%) and were married (78.1%). One third of the participants (33.5%) were of primary education or below, and around half had a total monthly household income of RMB 2000 or below (49.8%). Majority of them have been diagnosed with HIV positive for more than 5 years (83.1%), were in asymptomatic stage (78.8%), and had 2 children or less (75.5%). (Table 1)

**Table 1:** Background characteristics of the participants (*N* = 269)

	*N*	%
**Socio-demographic variables**		
**Age group**		
30 or below	24	8.9
31–40	158	58.7
41–50	78	29.0
51–60	4	1.5
61 or above	3	1.1
**Ethnicity**		
Han majority	239	88.8
Other minorities	30	11.2
Education level		
Primary or below	90	33.5
Secondary or above	179	66.5
**Marital Status**		
Married	210	78.1
Single/Others	59	21.9
**Monthly household income (RMB)**		
1000	48	17.8
1001–2000	87	32.3
> 2000	134	49.8
**Number of children**		
1	106	39.4
2	97	36.1
3	37	13.8
4 or above	29	10.8
**Age of the eldest child**		
5–10	100	37.2
11–20	156	58.0
> 20	13	4.8
**Gender of the eldest child**		
Male	140	52.0
Female	129	48.0
**Disease-related characteristics**		
**Duration of HIV diagnosis**		
5 years or below	45	16.9
6–10 years	90	33.7
> 10 years	132	49.4
**Self-reported route of HIV infection**		
Sexual transmission	244	90.7
Injective drug use	12	4.5
Don’t know/don’t wish to answer	13	4.8
**Disease stage**		
Asymptomatic	212	78.8
Symptomatic	8	3.0
AIDS	38	14.1
Uncertain	11	4.1
**On antiretroviral treatment (ART)**		
Yes	257	97.7
No	6	2.3

### Correlations between variables of interest

Table 2 shows the correlation between variables. Among the socio-demographic and medical variables, number of children (*r*=-.20, *P* <.01), age of the eldest children (*r*=-.18, *P* <.05) had a negative correlation, while duration of the HIV (*r* =.22, *P* <.01) had a positive correlation with participants’ intention to disclose their HIV status.

Furthermore, positive outcome expectations (*r* =.29, *P* <.01) had a positive correlation, while perceived health (*r*=-.15, *P* <.01) and negative outcome expectations (*r*=-.31, *P* <.01) had a negative correlation with intention to disclose HIV. These variables were entered in the subsequent hierarchical regression analysis.

**Table 2:** Correlation between variables

	1	2	3	4	5	6	7	8	9	10	11	12	13	14
1. Age	-													
2. Ethnicity (Ref: Han)	0.03	-												
3. Education level	0.04	0.10	-											
4. Marital status	0.16**	0.02	0.01	-										
5. Monthly household income	− 0.11	0.10	0.26**	− 0.17**	-									
6. Number of children	0.17**	− 0.12	− 0.24**	− 0.26**	− 0.08	-								
7. Age of the eldest child	0.51**	0.08	− 0.02	0.15*	− 0.11	0.04	-							
8. Gender of the eldest child (Ref: Male)	0.00	0.08	− 0.10	− 0.04	− 0.10	0.10	− 0.07	-						
9. Duration of HIV	0.16**	0.04	0.15*	0.03	− 0.02	− 0.18**	0.04	− 0.02	-					
10. On ART (Ref: No)	− 0.13*	− 0.03	0.00	− 0.11	0.10	0.05	− 0.09	− 0.11	0.10	-				
11. Perceived Health	− 0.21**	0.01	0.08	− 0.12	0.11	0.17**	− 0.15*	− 0.01	− 0.22**	0.02	-			
12. Positive outcome expectations	− 0.02	0.05	0.06	0.08	0.01	− 0.19**	0.02	− 0.01	0.03	− 0.09	− 0.06	-		
13. Negative outcome expectations	− 0.06	− 0.00	− 0.10	− 0.10	0.01	0.13*	0.03	− 0.01	− 0.11	0.02	-17**	− 0.02	-	
14. Intention to disclose HIV	0.00	− 0.11	0.06	− 0.03	− 0.03	− 0.20*	− 0.18**	0.05	0.22**	0.05	− 0.15*	0.29**	− 0.31**	-

* *P* <.05, ** *P* <.01, *** *P* <.001 ART: antiretroviral treatment

### Hierarchical regression model of intention to disclose HIV

Results from hierarchical regression analysis showed that among the socio-demographic and medical variables, ethnicity (*β*=-0.14, *P* <.01), number of children, (*β*=-0.23, *P* <.01), and duration of HIV (*β* = 0.24, *P* <.01) significantly predicted intention to disclose HIV. After adjusting for socio-demographic variables and medical variables, perceived health had no significant association with intention to disclose HIV when it was added in Block 2.

Furthermore, positive outcome expectations had a positive association (*β* = 0.41, *P* <.01), while negative outcome expectations had a negative association with intention to disclose HIIV (*β*=-0.21, *P* <.01) when they were added in Block 3. The overall model explained 28% of variance of intention to disclose HIV, F(12) = 7.45, *P* <.001 (Table 3).

**Table 3:** Hierarchical regression analysis of intention to disclose HIV

	Block1			Block 2			Block 3		
	B	*β*	VIF	B	*Β*	VIF	B	*Β*	VIF
Age	0.01	0.05	1.47	0.01	0.05	1.48	0.01	0.03	1.48
Ethnicity (Ref: Han)	0.06	0.01	1.08	0.05	0.01	1.09	0.01	0.00	1.09
Education level	0.16	0.07	1.15	0.17	0.07	1.17	0.05	0.02	1.21
Marital status	0.26	0.06	1.11	0.25	0.06	1.12	− 0.01	− 0.00	1.14
Monthly household income	− 0.09	− 0.06	1.15	− 0.08	− 0.05	1.18	− 0.11	− 0.07	1.19
Number of children	− 0.17	− 0.11	1.27	− 0.16	− 0.10	1.37	− 0.13	− 0.09	1.35
Age of the eldest child	− 0.29	− 0.13	1.51	− 0.30	− 0.13	1.56	− 0.17	− 0.07	1.56
Gender of the eldest child (Ref: Male)	− 0.04	− 0.02	1.17	− 0.05	− 0.02	1.1	0.09	0.04	1.20
Duration of HIV	0.36	0.23*	1.20	0.34	0.21*	1.30	0.22	0.14	1.33
On ART (Ref: No)	− 0.34	− 0.03	1.09	− 0.30	− 0.03	1.10	0.19	0.02	1.11
Perceived health				− 0.08	− 0.04	1.30	− 0.01	− 0.00	1.38
Positive outcome expectations							0.05	0.41***	1.07
Negative outcome expectations							− 0.02	− 0.21**	1.18
DF	10			11			13		
F	1.33			1.21			4.17***		
R square	0.10			0.10			0.31		

The overall model explained 28% of variance of intention to disclose HIV, F(12) = 7.45, *P* <.001 (Table 3). This corresponds to approximately a medium effect size (Cohen’s f2) of 0.45.

### Moderating role of perceived health on the association between outcome expectations and intention to disclose HIV

**Fig. 1:**
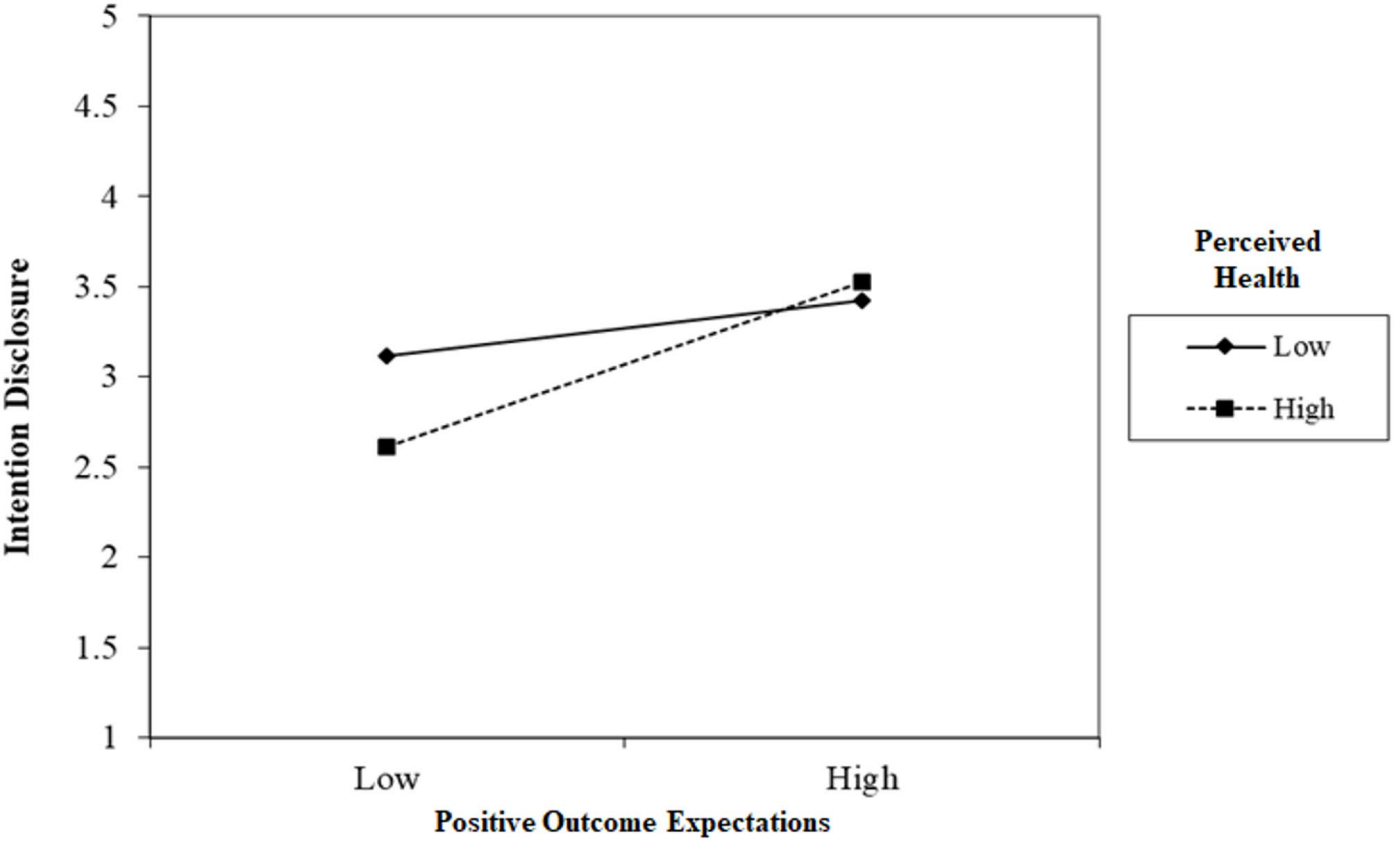
Interaction effect of positive outcome expectations and perceived health on intention to disclose HIV. (Note: 1 standard deviation below and 1 standard deviation above are used for low and high values respectively.)

Results of the hierarchical regression revealed a significant interaction term of positive outcome expectations and perceived health (*β* = 0.12, *P* <.05) in predicting intention to disclose HIV. However, the interaction term of negative outcome expectations and perceived health was not significant (Table 4). Further analysis showed that the association between positive outcome expectations and intention to disclose HIV was stronger among participants with better perceived health (Fig. 1).

**Table 4:** Interaction term of perceived health and outcome expectations on intention to disclose HIV

	B	β	VIF
**Model 1**			
Duration of HIV	0.26	0.16	1.08
Perceived health	− 0.11	− 0.10	1.06
Positive outcome expectations	0.32	0.27***	1.00
Interaction term of perceived health and positive outcome expectations	0.19	0.17**	1.02
**Model 2**			
Duration of HIV	0.23	0.14*	1.06
Perceived health	− 0.07	− 0.06	1.08
Negative outcome expectations	− 0.31	− 0.26***	1.04
Interaction term of perceived health and negative outcome expectations	− 0.03	− 0.02	1.00

* *P* <.05, ** *P* <.01, *** *P* <.001

For model 1, the R square increases from 0.14 to 0.17, with an R square change (ΔR²) for adding the interaction term between perceived health and positive outcome expectation is 0.03. This corresponds to a small to medium effect size (Cohen’s f2) of 0.20.

For model 2, there was no change in R square of 0.12 after adding the interaction term, corresponding to a relatively small effect size of 0.14.

## Discussion

This study examined the factors influencing intention to disclose HIV status to their children among HIV-infected women and examined the moderating role of perceived health on the association between outcome expectations and disclosure intention. Ethnicity, number of children, duration of HIV, positive outcome expectations, and negative outcome expectations were all significantly associated to HIV disclosure intention. Notably, a significant moderating effect of perceived health on positive outcome expectations was observed, indicating that the positive association between outcome expectations and disclosure intention was stronger among women with better perceived health.

The present study identified several important factors for HIV disclosure among women who have been diagnosed with the disease. Among the various socio-demographic and medical factors, the present study found that belonging to an ethnic minority and having more children were negatively associated with the intention to disclose their HIV status. Women from ethnic minority groups may face more challenges and social stigmatization when it comes to disclosing their HIV status, ultimately reducing their desire to disclose [29]. Similarly, women with more children may have additional responsibilities which can be a source of stress, thus reducing their willingness to reveal their HIV status [30]. Conversely, the duration of HIV had a positive association with the intention to disclose HIV. Women who had been living with HIV for an extended period may be more likely to have received counseling from healthcare professionals on self-care behaviors such as HIV disclosure, promoting their intention to disclose HIV [31].

The present study also provided empirical evidence that positive and negative outcome expectations are salient in women’s intention to disclose their HIV status to their children. Women who expect more positive outcomes of HIV disclosure would be more likely to have the intention to disclose in the future, while those holding negative attitudes about the outcomes of HIV disclosure would be less intended to do so. Findings are consistent to the extant literature and theories that anticipated outcomes predicted both disclosure of disease and the depth of the disclosure [14, 22, 23]. Furthermore, positive outcome expectations were found to have stronger association with intention to disclose than negative outcome expectations. Findings imply that HIV status disclosure decision making may involve the constant process of balancing risks and benefits, a compromise between individuals’ needs, and their concerns for self, others, and relationship with others [14]. We also identified a negative association between negative outcome expectations and HIV disclosure. Fear of stigma and family conflict are suggested as significant barriers to HIV disclosure [32, 33]. Stigma often arises from misinformation and societal stereotypes, leading to distressing emotions and social losses for both patients and their families. Additionally, concerns about rejection, privacy, and the potential for increased family tension are major factors that deter individuals from disclosing their HIV status. Future studies should incorporate qualitative research to gain a deeper understanding of these negative expectations among HIV-infected mothers to inform targeted interventions.

Disease progression theory demonstrates that medical, physical, and psychological demands aroused from deteriorating health would facilitate disclosure of HIV status [18]. Despite a significant negative correlation between perceived health status and intention to disclose HIV status, our study revealed that perceived health status was not a clear factor of HIV disclosure intention after taking account into sociodemographic and medical variables. In fact, studies have pointed out that poor health status not only motivates disclosure but also arouses fears about disclosure and help-seeking [34]. Therefore, health status should be considered together with other factors, rather than independently as it is associated with disclosure. Interestingly, the present study found a significant interaction effect between perceived health status and positive outcome expectations on HIV disclosure intention. Specifically, the effects of positive outcome expectations are found to be stronger among women with better perceived health. This finding highlights the importance of considering both psychological and health-related factors when designing interventions to support HIV status disclosure. According to Lazarus and Folkman’s stress and coping model, positive emotions can act as a buffer against stress [24]. Healthier individuals may experience less stress related to HIV disclosure and have more adaptive coping resources, which in turn can strengthen their positive outcome expectations.

### Implications

Findings of the present study provide important implications for practice. As people living with HIV are recommended to disclose their HIV status, interventions are needed to facilitate disclosure decision making. Findings of the study suggested that interventions that facilitate HIV disclosure should promote positive outcome expectations associated with HIV disclosure. Positive outcome expectations can be enhanced by multiple ways. For example, cognitive-behavioral therapy can help individuals identify and challenge negative thought patterns, replacing them with more positive and constructive alternatives [35]. Support groups can provide a platform for people to share experiences, receive encouragement, acquire information about the benefits of HIV disclosure, and learn from others who have successfully disclosed their HIV status [36]. Observing role models can also inspire and strengthen positive outcome expectations. Health care professionals should also seek to help women obtain an accurate appraisal of their ability in disclosure, which may help them feel more comfortable with self-disclosure and increase their intention towards it. Furthermore, findings suggest that it would be equally important to reduce negative outcome expectations of HIV disclosure. Counseling services should be provided to address any concerns or fears about societal stigma or discrimination, and work to reduce the possible negative consequences based on the patients’ current context.

### Limitations

There were several limitations of the study. First, the study was cross-sectional in nature so causality could not be assumed. Second, self-selection bias might exist and participants who took part in the study might have a more positive view towards HIV disclosure. Third, due to the cross-sectional nature of the study, it would be deemed inappropriate to examine the association between the variables of interests and past HIV disclosure behaviors. Therefore, only intention to disclose HIV was measured in the study. As advocated in the literature, intention-behavior gap exists. Fourth, we employed a single item to assess perceived health. Although single item is commonly used in research, it may oversimply the complex nature of this construct. Future studies should consider using a validated multi-item scale to provide a more comprehensive assessment of perceived health. Fifth, our study primarily focused on individual-level factors in HIV disclosure intention but did not account for the roles of social support or community-level influences, which also play critical roles in HIV disclosure. Future studies could consider integrating multiple levels of analysis to better understand the interplay between individual, social, and community factors in HIV disclosure. Finally, participants were only recruited from Yunnan and Guangxi provinces. The findings of the study might not be generalizable to women living with HIV in other regions in China. Future studies should consider recruiting a more geographically and socioeconomically diverse sample to enhance the external validity.

## Conclusion

In conclusion, the present study explored the relationship between outcome expectations and maternal HIV status disclosure intention among women living with HIV, and suggested that the association between positive outcome expectations and intention to disclose HIV was stronger among women with better health. Findings can inform the design of health interventions in empowering women with HIV to make informed decisions about HIV disclosure, which could bring beneficial outcomes to both mothers and their children.

## Data Availability

The datasets used and/or analysed during the current study are available from the corresponding author on reasonable request.

## Abbreviations

ART: Antiretroviral treatment
